# Mineralization and Detoxification of the Carcinogenic Azo Dye Congo Red and Real Textile Effluent by a Polyurethane Foam Immobilized Microbial Consortium in an Upflow Column Bioreactor

**DOI:** 10.3390/ijerph120606894

**Published:** 2015-06-16

**Authors:** Harshad Lade, Sanjay Govindwar, Diby Paul

**Affiliations:** 1 Department of Environmental Engineering, Konkuk University, Seoul 143-701, Korea; E-Mail: harshadlade@gmail.com; 2 Department of Biochemistry, Shivaji University, Kolhapur 416004, India

**Keywords:** Congo red, microbial consortium, immobilization, column bioreactor, decolorization, mineralization

## Abstract

A microbial consortium that is able to grow in wheat bran (WB) medium and decolorize the carcinogenic azo dye Congo red (CR) was developed. The microbial consortium was immobilized on polyurethane foam (PUF). Batch studies with the PUF-immobilized microbial consortium showed complete removal of CR dye (100 mg·L^−1^) within 12 h at pH 7.5 and temperature 30 ± 0.2 °C under microaerophilic conditions. Additionally, 92% American Dye Manufactureing Institute (ADMI) removal for real textile effluent (RTE, 50%) was also observed within 20 h under the same conditions. An upflow column reactor containing PUF-immobilized microbial consortium achieved 99% CR dye (100 mg·L^−1^) and 92% ADMI removal of RTE (50%) at 35 and 20 mL·h^−l^ flow rates, respectively. Consequent reduction in TOC (83 and 79%), COD (85 and 83%) and BOD (79 and 78%) of CR dye and RTE were also observed, which suggested mineralization. The decolorization process was traced to be enzymatic as treated samples showed significant induction of oxidoreductive enzymes. The proposed biodegradation pathway of the dye revealed the formation of lower molecular weight compounds. Toxicity studies with a plant bioassay and acute tests indicated that the PUF-immobilized microbial consortium favors detoxification of the dye and textile effluents.

## 1. Introduction

Azo dyes represents a major class of synthetic organic colorants that are extensively used for dyeing purposes in the textile industry because of their variety of shades, resistance to fading, excellent attachment to fabrics and lower energy consumption [1]. Azo dyes are characterized by the presence of one or more chromophoric azo (R_1_–N=N–R_2_) groups and aromatic rings, mostly substituted by sulfonate groups [2]. Azo dyes are highly stable to light, heat, water, detergents, bleach and perspiration due to their resonance and π-conjugated azo bond characteristics [3]. These properties make them highly suitable for use in the textile, paper, pharmaceutical, cosmetic and various chemical industries. Approximately 10,000 different dyes and pigments are produced commercially worldwide, with an annual production of 7 × 10^5^–1 × 10^8^ tons; among them nearly 60%–70% are azo dyes [4,5]. Following the extensive use for dyeing and finishing processes at various industries, a large volume of azo dye-containing wastewaters get discharged into the natural ecosystem. This poses a serious health hazard to humans and aquatic living creatures, as azo dyes are known to be toxic and their reductive cleavage products (aromatic amines) are mutagenic and even carcinogenic [6,7,8]. For instance, the diazo dye Congo red is known to be carcinogenic because of the presence of aromatic amine groups [9]. Additionally, the synthetic origin and complex aromatic structures of azo dyes make them highly recalcitrant to microbial degradation and thus they remain in the environment for a long period of time [10]. However, environmental legislation establishing limits on dye discharge has become more stringent and for human health concerns, typically obliges industries to remove dyes from their wastewaters before being released into water bodies.

Various physicochemical methods can be used to remove dyes from wastewaters such as photocatalysis, ozonation, membrane separation, electrochemical oxidation, activated carbon adsorption, coagulation and Fenton reagent oxidizer [2,11,12]. The most commonly used method is coagulation which is characterized by the rapid transfer of dyes from the liquid to the solid phase, but this requires high cost coagulants and produces a substantial amount of secondary waste [13]. Another method which is being used on a commercial scale is the use of low-cost adsorbents such as fly ash or activated carbon, but the need to dispose of dye-adsorbed waste limits its usefulness [14]. The Fenton reagent is an effective oxidizer, but this method produces a large amount of Fe(OH)_3_ precipitate as well as causes additional water pollution by the catalyst added as salt [13]. The overall treatment cost, extent of dye removal, feasibility, stability, residual sludge generation and generation of secondary pollutants with physicochemical methods are some of their inherent limitations [15]. Also, these methods may not completely mineralize or detoxify a wide variety of synthetic dyes under certain environmental conditions. In an effort to effectively remove the dyes from wastewaters and eliminate the challenges associated with physicochemical methods, researchers have investigated a variety of biological treatment strategies.

The biodegradation of dyes has shown better results and is emerging as an effective and promising approach for the mineralization and detoxification of azo dyes. This takes advantage of microorganisms to breakdown dyes and convert them into CO_2_ and H_2_O [16]. A wide spectrum of microorganisms, including bacteria and fungi, has been reported for the degradation of azo dyes [17,18,19] and different bioreactor studies have also been performed with pure cultures as well as their consortia [20,21]. It is worth mentioning that many of these studies were often performed with single cultures which are known to form toxic aromatic amines as azo dye degradation metabolites. Furthermore, single cultures are often specific to a specific type of dye, cannot mineralize a wide range of structurally different dyes and are difficult to scale up for large scale operations like RTE treatment plants, which limits their extensive use [21]. Alternatively, mixed cultures tend to possess enhanced decolorization activity and are more resistant to extreme pH (acidic or alkaline), temperature (high or low) and high dye concentrations [21,22]. In addition, mixed cultures could reduce the dye toxicity more satisfactorily than single cultures and are more suitable for large scale dye-containing wastewater treatment [23].

The immobilization of microbial cultures on carriers has widened their applications allowing them to be reused and improve their performance by providing greater stability towards a wide range of temperature and pH values and exposure to higher dye concentrations [24]. Additionally, immobilization can reduce the risk of biomass wash-out. These enhanced features have encouraged the use of immobilized cultures in a continuous reactor to treat large volumes of dye-containing wastewaters. To continually keep dye-degrading microorganisms in the reactor, it is necessary to select appropriate matrices for whole cell immobilization. Polyurethane foam (PUF) with macropores has attracted much attention as a most suitable support for immobilization of bacteria due to its high porosity, good mechanical strength, resistance to microbial attack, large surface area and easy availability at low-cost [25]. It not only retains the microorganisms, but also acts as a carrier for active biomass. Therefore, PUF was selected as a carrier for the immobilization of a microbial consortium. Recently, PUF has been used as carrier for the immobilization of bacteria and found to be an ideal carrier for the adhesion of bacterial cells, which were then tested for the removal of dye under anoxic reactor conditions [26].

The key factor which limits widespread application of bioreactors in dye removal is the high cost of the growth medium used. Most of the studies on dye-degrading bioreactors often use defined growth media such as nutrient broth, which is not affordable for use at commercial levels [27]. Alternatively, the use of agricultural waste as a growth medium has been suggested a low-cost alternative to make bioreactor use more economical [20,22]. Millions of tonnes of crop biomass and their by-products are generated as agricultural waste every year worldwide and are abundantly available at low-cost [28]. The use of agricultural waste wheat bran as growth medium for the enrichment of microbial consortia and their further application in the degradation of the carcinogenic azo dye Trypan blue at the flask level has been reported [22]. Looking towards the ability of microbial consortia to grow in wheat bran medium and effectively degrade and detoxify carcinogenic azo dyes within a short time, the present study considered three strategies for dye-containing wastewater treatment: (1) development of a microbial consortium that could decolorize the model carcinogenic azo dye CR (2) complete mineralization and detoxification of CR dye and RTE; and (3) development of a PUF-immobilized microbial consortium upflow column bioreactor for the mineralization of dyes.

## 2. Materials and Methods

### 2.1. Azo Dye, RTE, Chemicals and WB Medium

The diazo dye Congo red (C.I. No. 22120, Direct red 28, MF: C_32_H_22_N_6_Na_2_O_6_S_2_ and FW: 696.66 g·mol^−1^) which is the sodium salt of benzidinediazo-bis-1-naphthylamine-4-sulfonic acid, used in this study was supplied by Sigma-Aldrich (St. Louis, MO, USA). RTE was collected from Mahesh Textile Processors (Ichalkaranji, MS, India) in an airtight plastic can, passed through Whatman grade No. 1 filter paper to remove large suspended matter and stored in a refrigerator at 4 ± 1 °C until further use. Veratryl alcohol, catechol, methyl red, 2,2′-azino-bis (3-ethylbenzothiazoline-6-sulfonic acid) (ABTS), nicotinamide adenine dinucleotide (NADH), dichlorophenol indophenol (DCIP), glucose, yeast extract and peptone were purchased from HiMedia Laboratories Pvt. Ltd. (Mumbai, MS, India). All of the chemicals used in this study were of analytical grade, unless otherwise mentioned. WB medium was prepared, the pH adjusted to 7.5 with with 0.1 N NaOH or 0.1 N HCL and used as growth medium for the biodegradation of dye and textile effluent [22].

### 2.2. Development of Dye CR Decolorizing Microbial Consortium

Soil samples were taken from the top 10 cm layer in the wastewater disposal site of textile processing units located on an industrial estate area (Ichalkaranji, MS, India). The collected soil was transported to the laboratory in an airtight polythene bag and stored at 4 ± 1 °C until further use. To obtain the dye CR decolorizing microbial consortium, 1.0 gm of soil sample was inoculated into a 250 mL Erlenmeyer flask that contained 100 mL of WB medium amended with 100 mg·L^−1^ of CR. This flask was incubated under microaerophilic conditions (*i.e.*, no aeration and agitation) at 30 ± 0.2 °C for enrichment. After 7 days, an aliquot (about 5 mL) was transferred to 100 mL of fresh WB medium supplemented with same amount of dye CR (100 mg·L^−1^) and incubated under similar conditions. The consecutive acclimatization of enriched microbial consortium was carried out until complete decolorization of CR dye observed. This developed microbial consortium was then maintained in WB medium in the presence of 100 mg·L^−1^ CR dye at 4 ± 1 °C for routine use.

### 2.3. Bacterial Diversity Analysis of the Developed Microbial Consortium

The developed microbial consortium was analyzed for the presence of bacterial diversity by polymerase chain reaction-denaturing gradient gel electrophoresis (PCR-DGGE) as described earlier [29]. For this, the developed microbial consortium was overnight grown in WB medium containing CR dye (100 mg·L^−1^) at 30 ± 0.2 °C and genomic DNA was extracted [30]. The 16S ribosomal RNA gene was amplified by PCR using the forward primer RDB1-GC clamped (F58 CGCCGCCGCGCCCCGCGCCCGGCCCGCCGCCGCGGCCGCAGTTTGATCCTGGCTCA) and reverse primer RDB2 (GGACTACCAGGGTATCTAAT). The primers, enzymes, and slats from PCR amplicons were removed by PureLink PCR purification kit (Invitrogen, Bedford, MA, USA) and the DGGE was performed using a D-code System (Bio-Rad Laboratories, Singapore) at 60 °C in 1 × TAE buffer [31]. The PCR products were loaded onto 8% (*w/v*) polyacrylamide gels (37.5:1, acrylamide/bisacrylamide) using a denaturing gradient ranging from 30% to 55% denaturant, run at 75 V and 60 °C for 16 h, stained with silver nitrate and visualized.

### 2.4. Preparation of Cell Free Extract and Enzyme Assays

The developed microbial consortium was pre-grown in 250 mL Erlenmeyer flasks containing 100 mL of WB medium (pH 7.5) at 30 ± 0.2 °C for 24 h under microaerophilic conditions. The enriched consortium was then used to decolorize CR dye (100 mg·L^−1^) and RTE (50% in WB medium) under similar conditions. After complete decolorization, the cell free extract of the enzymes were prepared as per an earlier report [22]. A similar procedure was followed with the pre-grown microbial consortium culture broth before addition of dye or textile effluent to obtain initial enzyme activities.

The activities of oxidative and reductive enzymes were assayed spectrophotometrically. Laccase activity was determined in a 2 mL reaction mixture by monitoring the oxidation of 1 mM ABTS in sodium acetate buffer (100 mM, pH 4.9) at 420 nm [32]. Tyrosinase activity was measured at 495 nm by observing the formation of catechol quinone in a reaction mixture [33]. Veratryl alcohol oxidase activity was determined by monitoring the oxidation of 1 mM veratryl alcohol in citrate phosphate buffer (100 mM, pH 3.0) at 310 nm due to the formation of veratraldehyde [34]. Azoreductase activity was estimated by monitoring the absorbance decrease of methyl red at 430 nm [35]. DCIP reductase activity was calculated by the reduction of DCIP at 590 nm [36]. All the enzymatic reactions were carried out at room temperature (30 ± 1 °C) where reference blanks contained all components expect enzyme. All assays were run in triplicate and the average rates were calculated to represent the enzyme activity. The protein content was determined based on Lowry *et al.* method with bovine serum albumin as standard [37].

### 2.5. Immobilization of Developed Microbial Consortium on PUF

The PUF was cut into 1-cm^3^ cubes, washed twice with distilled water and oven-dried at 37 °C followed by autoclave sterilization at 121 °C for 15 min in a 250 mL Erlenmeyer flask [26]. The developed microbial consortium was grown in WB medium at 30 ± 0.2 °C for 24 h under microaerophilic conditions, cells were harvested (7500× *g* for 15 min, 4 ± 0.2 °C) and used for immobilization purpose. For this, 100 mg of microbial consortium cells were added to 100 mL fresh WB medium (pH 7.5) and transferred to the flask containing 1 gm of sterilized PUF cubes. This content was incubated at 30 ± 0.2 °C for 24 h under shaking conditions (120 rpm) to allow the anchoring of bacterial cells on PUF surface. Finally, the medium was removed and microbial consortium-anchored PUF cubes were washed with steriled saline under clean bench to remove loosely bound bacteria and stored at 4 ± 1 °C for further decolorization experiments.

### 2.6. Batch Decolorization Experiments with PUF-Immobilized Consortium

To study the effect of operating conditions on decolorization efficiency, batch experiments were carried out in 250 mL Erlenmeyer flasks containing 100 mL of WB medium and 1 gm of PUF-immobilized microbial consortium. Initially, dye CR (100 mg·L^−1^) was added into PUF-immobilized culture flask and the effect of microaerophilic and aerobic (agitation at 120 rpm) incubation on decolorization was evaluated at 30 ± 0.2 °C. Further decolorization studies were carried over a range of pre-grown culture medium pH (4–10), incubation temperatures (25, 30, 37 and 40 ± 0.2 °C) and initial dye concentrations (50–200 mg·L^−1^). An aliquot (3 mL) of culture medium was withdrawn at pre-determined time intervals, cell biomass was separated by centrifugation (7500× *g* for 15 min, 4 ± 0.2 °C) and the absorbance of resulted supernatant was measured on an UV-VIS Spectrophotometer (UV-1700 Model, Shimadzu, Kyoto, Japan) at λ_max_ = 497 nm. The percentage of dye decolorization was then calculated as follows:

(1) $$\text{Decolorization (\%) = }\frac{\text{Initial absorbance}(0h)\;-\text{ Final absorbance}(t)}{\text{Initial absorbance}(0h)}\times 100$$

Furthermore, the batch decolorization of RTE using PUF immobilized microbial consortium (1 gm) was investigated under microaerophilic and aerobic incubation conditions as well as at different RTE concentrations v.z. 25%, 50% and 75% in WB medium. The similar experimental conditions used for dye CR decolorization were adopted for RTE *i.e.*, WB medium pH 7.5 and incubation temperature 30 ± 0.2 °C unless otherwise stated. The dilutions of RTE were prepared using double strength WB medium (*i.e.*, 10 gm per 100 mL of wheat bran extract) and distilled water in such a way that the final composition of culture medium remains single strength. The ADMI removal values were used to calculate the decolorization of textile effluent. All the experiments were conducted at least in triplicate by changing one parameter at a time while other kept constant. Erlenmeyer flask containing 1 gm PUF cubes (without microbial consortium) served as abiotic control.

### 2.7. Effect of Additional Carbon and Nitrogen Source on Decolorization of CR Dye and RTE

The effect of additional carbon and nitrogen sources on dye and textile effluent decolorization by PUF immobilized microbial consortium was investigated using flask studies. For this, 100 mL of WB medium was placed in the 250 mL Erlenmeyer flask and individually supplemented with 0.5 g·L^−1^ of glucose, yeast extract, peptone and their combinations [38]. One gram of PUF-immobilized microbial consortium was then added into each modified WB medium flasks and tested for decolorization of CR dye (100 mg·L^−1^) and RTE (50%) at 30 ± 0.2 °C under microaerophilic incubation conditions. CR dye was directly added into the WB medium, while RTE was mixed with an equal volume of double strength WB medium to make 50% dilution and tested. All the experiments were performed in triplicate and the average values were determined.

### 2.8. Upflow Column Bioreactor Development

**Figure 1:**
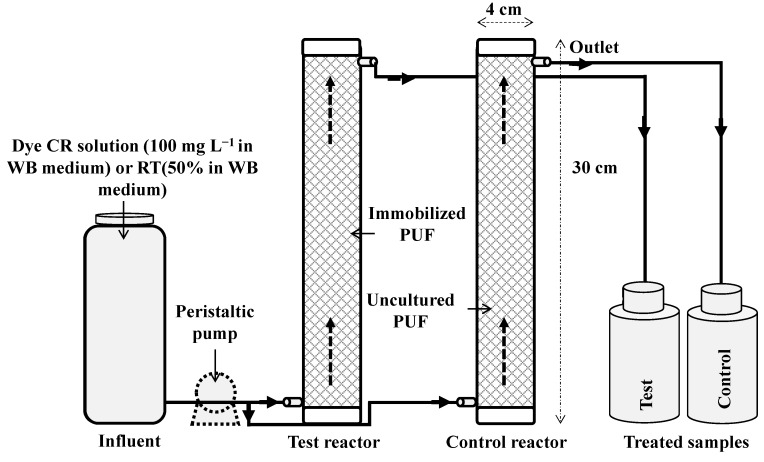
Schematic diagram of the developed upflow column bioreactor.

Glass columns (4 × 30 cm, total volume 200 mL) with a void volume of 50 mL were used for reactor development. The glass columns, silicon tubing and other accessories were autoclaved and connected aseptically. Microbial consortium-immobilized PUF cubes were filled into the column (20-cm bed) which served as test reactor, while a column filled with uncultured PUF cubes (without microorganisms) served as control reactor. The purpose of the control reactor was to confirm that the removal of dyes was not due to adsorption onto the PUF surface. The column reactors were installed as shown in Figure 1. Decolorization experiments for treatment of CR dye (100 mg·L^−1^ in WB medium) and RTE (50% in WB medium) with continuous upward feeding were conducted at various flow rates (5 to 50 mL·h^−1^) while keeping other parameters such as WB medium pH 7.5 and laboratory ambient temperature 30 ± 0.2 °C unchanged. The hydraulic retention time (HRT) was determined by dividing the effective volume of bioreactor by the influent flow rate. The as treated samples were collected from the outlet of the reactors and the percentage of dye decolorization and textile effluent ADMI removal measured. After optimizing the flow rates for enhanced decolorization performance, the upflow column bioreactors were again operated under optimum conditions and the mineralization of dye and textile effluent were determined. Decolorized CR dye samples were further analyzed using analytical techniques to reveal the biotransformation mechanism.

### 2.9. Characterization of Dye and Textile Effluent

The treated dye and textile effluent samples were subjected to TOC determination by a Sievers 5310C automated analyzer (GE Water & Process Technologies, Boulder, CO, USA), COD was determined by the dichromate closed reflux titrimetric method and BOD by a 5-day incubation test [39]. Similar procedures were also followed for untreated CR dye and RTE to obtain initial values. Both the bioreactors were operated in laboratory at constant temperature (30 ± 0.2 °C).

### 2.10. Extraction and Analysis of Dye CR Biotransformation Metabolites

The extraction and sample preparation of CR dye metabolites after PUF-immobilized microbial consortium treatment under upflow column bioreactor conditions was carried out [21]. For this, the treated dye samples were collected and suspended solids were removed by centrifugation (10,000× *g* for 15 min, 4 ± 0.2 °C). This resulted clear supernatant was extracted thrice with an equal volume of ethyl acetate, organic layer collected and dried in a SpeedVac (Thermo Fisher Scientific Inc., Waltham, MA, USA). It was then dissolved in methanol, filtered through 0.45 µm pore size cellulose acetate filters and subjected to HPLC (Agilent 1200 series HPLC system, Agilent Technologies, Santa Clara, CA, USA), FTIR (Nicolet iS 10 spectrometer, Thermo Fisher Scientific Inc., Waltham, MA, USA) and GC-MS (QP2010 GC-MS system, Shimadzu Corporation, Kyoto, Japan) analysis as per an earlier report [22]. CR dye (100 mg·L^−1^) was dissolved in methanol and analyzed to obtain control spectra.

### 2.11. Toxicity Analysis

The phytotoxicity testing of CR dye solution (100 mg·L^−1^), RTE (50%) and their PUF-immobilized microbial consortium-treated samples under upflow column bioreactor conditions was carried out at room temperature (30 ± 1 °C) [19]. The treated samples were further analyzed using an *in vitro* acute toxicity assay using *Daphnia magna* as model mammal [40]. For this, the samples treated in the upflow column bioreactor were centrifuged (7500× *g* for 20 min, 4 ± 0.2 °C), the clear supernatant collected and sterilized by filtration using 0.45 µm pore size cellulose acetate membrane filters. One hundred mL of these filter-sterilized samples as well as untreated CR dye solution (100 mg·L^−1^) and RTE (50%) were separately placed in 250 mL Erlenmeyer flasks and ten *D. magna* neonates (24 h old) were added to each flask. After incubation of 48 h at 20 ± 0.2 °C in dark, the numbers of immobile organisms were counted after exposing to light for 20 s. Both the toxicity tests were performed in triplicate with distilled water as control and average values calculated.

### 2.12. Statistical Analysis

The statistical analyses were performed using the program SPSS ver. 17.0 (SPSS, Chicago, IL, USA). Significance of variance was analyzed by one-way ANOVA with Tukey-Kramer multiple comparison test.

## 3. Results and Discussion

### 3.1. Microbial Community Analysis

**Figure 2:**
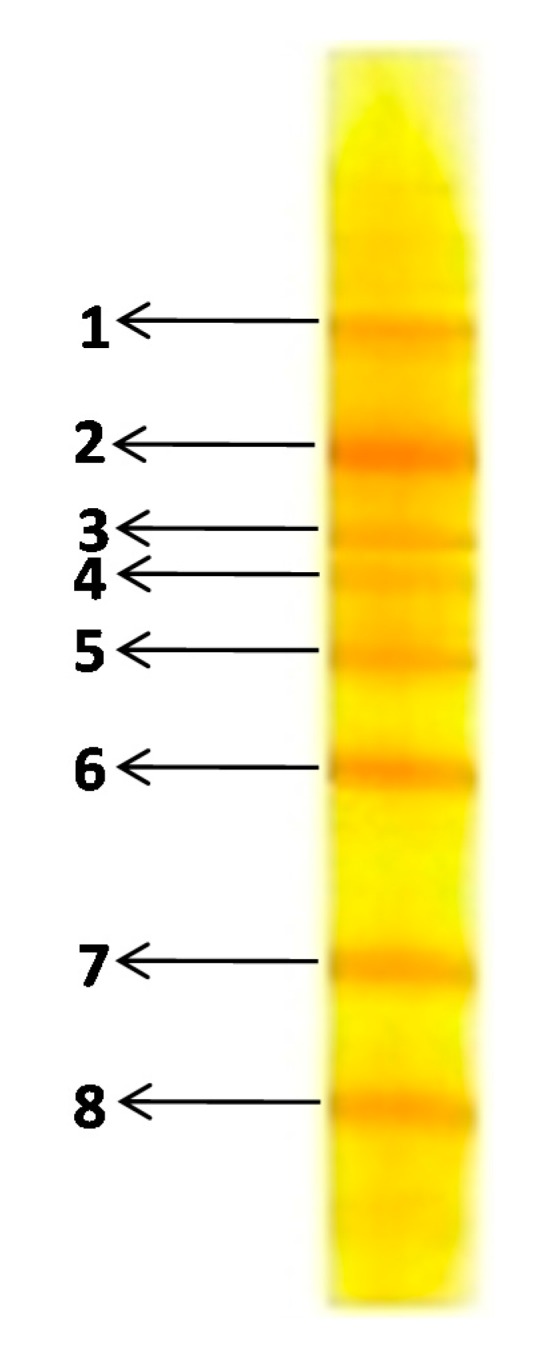
PCR-DGGE profile of microbial consortium.

As there are well-established concerns about the potential health hazards caused by azo dyes, a microbial consortium that could decolorize the model azo dye CR was enriched from textile wastewater disposed soils. The microbial diversity of the enriched microbial consortium was analyzed to demonstrate the metabolic potential and ecological functions of the textile wastewater disposal soil inhabitants. The microbial communities of contaminated sites are known to be responsible for overall metabolism and thus remediation of pollutants present. Many studies have attempted to explore the correlation between number of microbes present in textile wastewater dump sites and their dye degradation potential by means of DGGE analysis [22,29,31]. DGGE separates the PCR amplified DNA of the same size but different sequences and therefore each band formed in the denaturant gradient gel reveals the presence of a single bacterium [41,42]. The results of the PCR-DGGE analysis of 16S rRNA sequences revealed a total of eight distinctive bands, suggesting the presence of eight different bacteria in the enriched consortium (Figure 2). A study on the degradation of coagulated textile dyes sludge under solid state fermentation conditions also indicated that the developed microbial consortium from highly polluted textile industry sites possess 10 different bacteria as analyzed by DGGE [29]. Similarly, a microbial consortium enriched from dye contaminated soil that could degrade the azo dye Trypan blue has revealed the presence of 16 different bacteria [22]. Here, the main purpose of DGGE analysis was to estimate the number of bacteria present in te developed consortium that could grow in WB medium and decolorize the dye. Such a microbial consortium could be used under non-sterile conditions for the mineralization and detoxification of dyes; thus the bacterial identification part has been not incorporated in the current investigation.

### 3.2. Decolorization of Dye CR and RTE in WB Medium

While numerous studies on the removal of azo dyes by biodegradation exist, there is a lack of information on the use of agricultural wastes as microbial growth media [1,17,19,27]. Agricultural waste has begun to attract attention as microbial growth media as a solution to the high cost of routinely used defined growth media. Recently, the widely generated and abundantly available major agricultural waste wheat bran has been used as the growth medium for the enrichment of microbial consortia and their use in the biodegradation of the benzidine-based azo dye Trypan blue [22]. Since wheat bran has been previously found suitable for the growth of microorganisms and their use in the degradation of azo dyes at flask studies [22]; it has now been further evaluated with a PUF-immobilized microbial consortium for the mineralization and detoxification of the model azo dye CR and RTE under batch and laboratory scale continuous upflow column bioreactor conditions.

**Figure 3:**
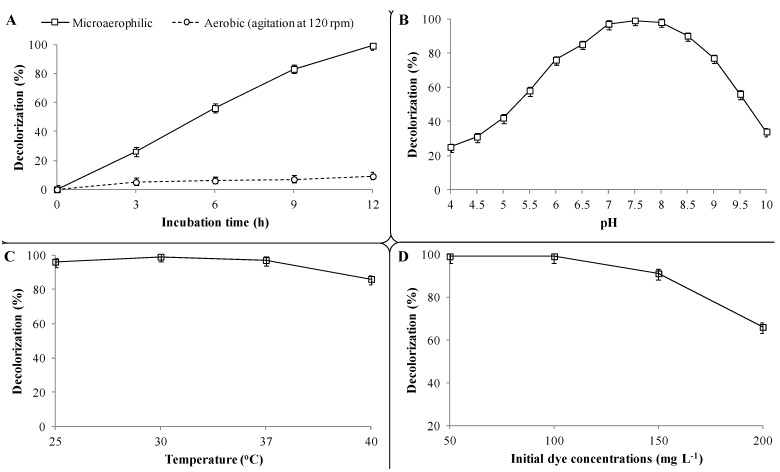
Batch studies on the effect of operational variables on the decolorization of CR dye by a PUF-immobilized microbial consortium. (**A**) Microaerophilic and aerobic incubation; (**B**) pre-grown culture medium Ph; (**C**) incubation temperatures and (**D**) initial CR dye concentrations. The percent decolorization was measured after 12 h of incubation at 30 ± 0.2 °C unless otherwise stated. Data points indicate the mean of three independent replicates; ±Standard errors of mean (SEM) is indicated by error bars.

Results of the batch experiments with the PUF-immobilized microbial consortium indicated that microaerophilic incubation favors CR decolorization as 99% of dye (100 mg·L^−1^) removal was observed within 12 h at 30 ± 0.2 °C (Figure 3A). On the contrary, only 9% dye removal was achieved within the same time and a maximum of 14% after 48 h under aerobic incubation conditions. The microbial decolorization of azo dyes is an enzymatic reaction and the presence of molecular oxygen has a strong inhibitory effect on dye removal [43] primarily due to competition in the oxidation of reduced electron carriers like NADH with either oxygen or azo groups as the electron receptor [44]. This perception has been well supported in a previous study, where oxygen-rich conditions had a strong inhibitory effect on the decolorization of the diazo dye Reactive blue 160 by mixed BDN cultures as only 24% dye removal was observed within 4 h, whereas more than 90% removal was achieved within same time under microaerophilic conditions [45]. Therefore, microaerophilic incubation conditions are necessary for azo dye reduction and thus further CR azo dye decolorization experiments with the PUF-immobilized microbial consortium were only performed using microaerophilic incubations.

The effect of different pre-grown culture medium pH on CR dye (100 mg·L^−1^) decolorization by the PUF-immobilized microbial consortium showed that the maximum dye removal (99%) was achieved at pH 7.5 within 12 h (Figure 3B). Further increases or decreases in culture medium pH towards slightly basic (7.0) or alkaline (8.0) conditions showed marginal decreases in dye removal (97% and 98%, respectively) within the same timeframe, whereas extreme acidic (4–6) or alkaline (9.0–10) pH values strongly retarded the decolorization performance (25% and 34%, respectively). Although the immobilized microbial consortium was active at extreme acidic and alkaline pHs, the enzyme activities might not be induced sufficiently to achieve optimum dye removal. The original pH of the pre-grown microbial consortium culture used in this study was 7.55 (without any pH adjustment). These results are in good agreement with a study where a microbial consortium composed of 15 different bacteria was proficient in the decolorization of the diazo dye Trypan blue (50 mg·L^−1^) at pH 7.0, while a slight decrease (6.0) or an increase (8.5) in pH did not affect decolorization rates much [22]. These results indicated that the PUF-immobilized microbial consortium was able to decolorize the azo CR dye in a basic to alkaline pH range and thus could be used for treatment of dye-containing wastewaters with variable pH ranges. It is known that textile effluents have a characteristic alkaline pH range [46].

Decolorization at different incubation temperatures revealed that the PUF-immobilized microbial consortium was able to remove the CR dye (100 mg·L^−1^) over a wide range of temperatures *i.e.*, 25–40 ± 0.2 °C. However, maximum dye removal (99%) was observed at an optimum incubation temperature of 30 ± 0.2 °C within 12 h, while 96%, 97% and 86% removal was achieved at 25, 37 and 40 ± 0.2 °C temperatures, respectively, within same time (Figure 3C). The same results were reported by Kadam *et al.* for the decolorization of the diazo dye Reactive rd 120 by a developed microbial consortium consisting of 10 different bacteria, which exhibited maximum dye removal at 30 ± 0.2 °C incubation temperature [29]. A previous study also showed that the enriched microbial consortium gave the maximum decolorization at 30 ± 0.2 °C while incubation at lower and higher temperatures than the optimum marginally affected decolorization rates [22]. The incubation temperature affects both microbial growth and enzyme activities, and thus the rate of dye decolorization [47]. Beyond the optimum temperature, the loss of cell viability and decreased rates of enzymes production occur, which result in reduced azo dye decolorization [2]. Thus, it was clear that, though the maximum dye decolorization often corresponds to the optimum incubation temperature, the PUF-immobilized microbial consortium was well adapted to a wide range of temperatures *i.e.*, 25 to 40 ± 0.2 °C for efficient CR dye decolorization.

Textile wastewaters are known to contain structurally different and varied concentrations of dyes ranging from 10 to 200 mg·L^−1^ [48]. Therefore, the microorganisms employed for decolorization of dyes-containing wastewaters must be able to tolerate high concentrations of dyes and should be metabolically active to mineralize them. The result of the decolorization at different dye concentrations showed that a maximum 100 mg·L^−1^ of dye CR was efficiently decolorized by the PUF-immobilized microbial consortium within 12 h (Figure 3D). Further increases in dye concentration up to 150 mg·L^−1^ slightly reduced the decolorization rate as 91% removal was observed within 12 h. However, at 200 mg·L^−1^ of CR dye concentration, a strong inhibition of decolorization was observed and only 66% removal was achieved within 12 h and as much as 20 h were needed for complete decolorization (data not shown). The ability of PUF-immobilized microbial consortium to decolorize a maximum of 100 mg·L^−1^ of CR dye in agricultural waste WB medium thus indicated its great potential to be used for treatment of azo dye-containing wastewater at low-cost.

**Figure 4:**
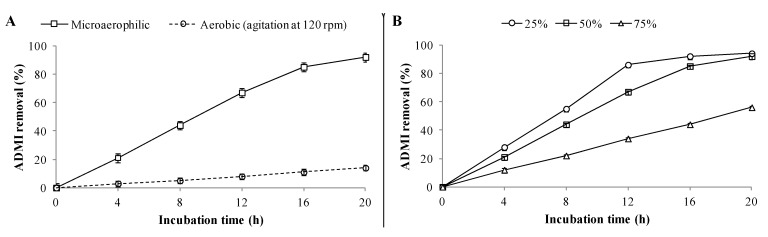
Batch studies on decolorization of RTE by PUF-immobilized microbial consortium at 30 ± 0.2 °C. (**A**) Microaerophilic and aerobic incubation; and (**B**) different RTE concentrations v.z. 25%, 50% and 75%. Decolorization was measured in terms of percent ADMI removal. Data points indicate the mean of three independent replicates; ±SEM is indicated by error bars.

Additionally, batch studies on the decolorization of RTE (50% in WB medium) using PUF-immobilized microbial consortium were also carried out, which showed 92% ADMI removal within 20 h under microaerophilic incubation conditions (Figure 4A), whereas only 12% ADMI removal was observed under aerobic incubation and a maximum of 16% within 48 h (data not shown). Thus, it would be essential to use microaerophilic conditions to achieve enhanced decolorization rates of textile effluents. The reduced decolorization performance under aerobic incubation conditions might be due to the presence of higher amounts of azo dyes in the RTE as the chromophoric azo groups of azo dyes get cleaved by azoreductase only in the absence of or with a limited supply of oxygen [49]. The decolorization results for different RTE concentrations revealed that a maximum of 94% and 92% ADMI removal were achieved for 25% and 50% diluted RTE respectively within 20 h by PUF-immobilized microbial consortium (Figure 4B). However, decolorization at higher RTE concentration (75%) was strongly inhibited as only 56% AMDI removal was achieved within 20 h and a maximum of 62% in 48 h (data not shown). Since the textile effluent treatment goal is to remove dyes, the maximum ADMI removal value obtained in this experiment (92% ADMI removal at 50% RTE) by PUF-immobilized microbial consortium was within the acceptable limits (≤150 ADMI units) set for the final discharge of effluent to streams or water bodies [50].

### 3.3. Effect of Additional Carbon and Nitrogen Sources on Decolorization

In an attempt to enhance the dye decolorization potential of the PUF-immobilized microbial consortium, the WB medium was supplemented with additional carbon and nitrogen sources and investigated under optimized conditions. The PUF-immobilized microbial consortium showed complete decolorization of CR dye and 92% ADMI removal of RTE in WB medium (without additional carbon or nitrogen source) within 12 and 20 h, respectively, under microaerophilic conditions. The decolorization result with glucose as an additional carbon source reveled that complete decolorization of CR dye and RTE (93% ADMI removal) was achieved within 11 and 19 h respectively (Table 1). The slightly enhanced decolorization rate after addition of glucose may be due to the fact it acts as a source of electron donors, which are needed for the cleavage of azo bonds [23,51]. It was also observed that addition of yeast extract and peptone was ineffective as complete decolorization of CR dye and RTE occurred in same time. Yeast extract (growth factor) or peptone (nitrogen source) in the decolorization medium is known to activate the coenzyme required for the metabolic pathway of azoreductase and thus serve as key component for enhanced azo dye decolorization [49].

When glucose was added in combination with yeast extract and peptone, the decolorization performance of the PUF-immobilized microbial consortium increased marginally compared to the individual carbon or nitrogen source-containing WB medium and the complete removal of dye CR and RTE was observed within 10 and 18 h, respectively. The reason for the marginal enhanced decolorization rate in the presence of combined carbon and nitrogen sources can be attributed to the enzyme-stimulatory activity of such treatment, which has been reported earlier [52]. The overall results suggested that WB medium without addition of supplementary carbon or nitrogen sources was sufficient for the growth of the microbial consortium and subsequent decolorization of dye and effluent. This highlights the potential of WB medium to be used for large scale treatment of dye-containing wastewaters. The real market cost analysis of agricultural waste wheat bran has already underlined the low-cost of WB medium [22].

**Table 1:** Effect of additional carbon and nitrogen sources on the decolorization of dye CR and RTE by PUF immobilized microbial consortium.

Medium	Dye CR (100 mg·L^−1^)		RTE (50%)	
	Decolorization (%)	Time (h)	ADMI Removal (%)	Time (h)
WB	99 ± 1.0	12	92 ± 1.0	20
WB + Glucose	99 ± 1.0	11	93 ± 1.0	19
WB + Yeast extract	99 ± 1.0	11	94 ± 1.0	19
WB + Peptone	99 ± 1.0	11	94 ± 1.0	19
WB + Glucose + Yeast extract	99 ± 1.0	10	95 ± 1.0	18
WB + Glucose + Peptone	99 ± 1.0	10	95 ± 1.0	18

Values are a mean of three experiments ±SE.

### 3.4. Upflow Column Bioreactor Study

**Figure 5:**
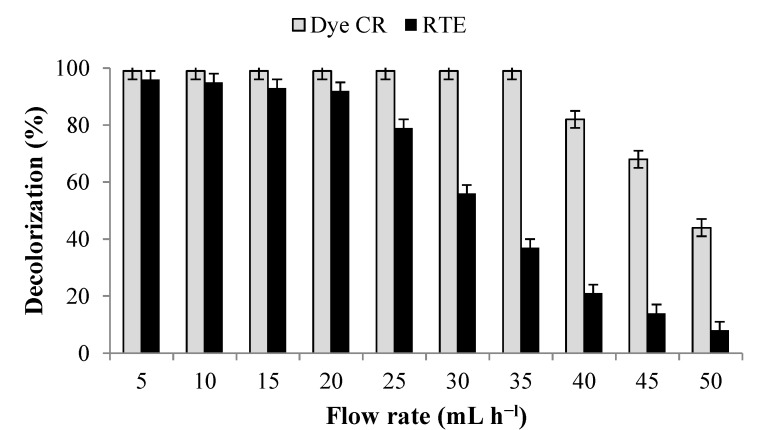
Decolorization of CR dye (100 mg·L^−1^) and RTE (50%) by PUF-immobilized microbial consortium under upflow column bioreactor conditions at various flow rates. Data points indicate the mean of three independent replicates; ±SEM is indicated by error bars.

To remove the dyes from a substantial amount of wastewater generated from the textile and dyestuff industries, there is a need for a continuous treatment process like immobilized microbial culture column reactors. Recently single culture immobilized bioreactors for the treatment of azo dyes and textile effluents under continuous operation using *Luffa cylindrica* as the immobilization material were reported [20,53]. However, single culture could not remove the mixture of structurally different dyes presents in the textile effluents as compared to microbial consortia [19], while, the mixed culture immobilized bioreactors are considered to represent a high rate continuous process suitable for the treatment of large amounts of dye-containing wastewaters [21]. Hence, in the present study a PUF-immobilized microbial consortium consisting of eight different bacteria has been investigated for the mineralization of azo dye and textile effluent under upflow column bioreactor conditions. The results of the decolorization of CR dye (100 mg·L^−1^) and RTE (50% in WB medium) at different volumetric flow rates revealed that maximum dye removal was obtained at 35 mL·h^−l^ (HRT 5.7 h) and 20 mL·h^−l^ (HRT 10 h) respectively (Figure 5). A further increase in flow rates beyond the optimum strongly inhibited the decolorization performance. The decolorization of both dye and textile effluent in a control reactor that contained uncultured PUF cubes was negligible and hence the dye removal with PUF immobilized microbial consortium was not because of adsorption by the PUF bed.

The analysis of TOC, COD and BOD of the dye and textile effluent before and after treatment was performed in order to confirm their mineralization. The dye decolorized at the optimized volumetric flow rate of 35 mL·h^−l^ and HRT 5.7 h showed 83%, 85% and 79% reduction in TOC, COD and BOD contents, respectively (Table 2). In the case of RTE (50%) treatment, 92% ADMI removal was observed with 79%, 83% and 77% reduction in TOC, COD and BOD content at a flow rate of 20 mL·h^−l^ and HRT 10 h. The high COD removal efficiency suggested the potential of the developed upflow column bioreactor for the mineralization of azo dyes and real textile effluents. The performance of textile effluent treatment processes has been known to be directly associated with removal of COD [54]. The enhanced reduction in TOC, COD and BOD contents indicates the efficient biodegradation of the CR dye and RTE in the column bioreactor by the immobilized microbial consortium.

**Table 2:** Characterization of CR dye and RTE before and after treatment with PUF-mmobilized microbial consortium under upflow column bioreactor conditions.

Parameters	Dye CR (100 mg·L^−1^)		RTE (50%)	
	Untreated	Treated (35 mL·h^−l^)	Untreated	Treated (20 mL·h^−l^)
TOC (mg·L^−1^)	1750 ± 5.0	295 ± 3.0	1887 ± 6.0	390 ± 4.0
COD (mg·L^−1^)	1325 ± 3.0	195 ± 1.0	1620 ± 4.0	275 ± 2.0
BOD (mg·L^−1^)	210 ± 2.0	44 ± 1.0	338 ± 3.0	78 ± 1.0
Color (%)/ADMI	100 ± 0.0	1 ± 1.0	1943 ± 10.0	150 ± 2.0

Values are a mean of three experiments ±SEM.

### 3.5. Enzymatic Analysis in Free Microbial Consortium Culture

The potential of enzymes like laccases, tyrosinase, veratryl alcohol oxidase, azoreductase and NADH-DCIP reductase in the decolorization and degradation of dyes has been well documented [55]. Several bacteria are known to possess various oxidative and reductive enzymes that can break down dyes into low molecular weight metabolites [55]. In the present study, the oxidative enzymes laccase (275%), tryrosinase (110%) and veratryl alcohol oxidase (46%) were found to be induced significantly in CR dye (100 mg·L^−1^) samples (12 h) decolorized by the developed microbial consortium when compared with control (Table 3). It was also observed that the reductive enzymes azoreductase (700%) and DCIP reductase (329%) showed higher induction in decolorized samples. The significant increase in both oxidative and reductive enzyme activities in decolorized broth indicated their possible involvement in the metabolism of CR dye. The same observation was reported by Lade *et al.* [22] that increase in oxidoreductive enzyme activities in the microbial consortium culture was observed when exposed to the azo dye Trypan blue in microaerophilic conditions. In the case of RTE (50%), a significant induction in the activities of all studied enzymes *i.e.*, laccase (350%), tryrosinase (170%), veratryl alcohol oxidase (77%), azoreductase (850%) and DCIP reductase (376%) was observed after complete decolorization (20 h) by the microbial consortium. The overall enzymatic analysis results revealed that different sets of oxidoreductive enzymes were significantly induced in the microbial consortium when exposed to CR dye (100 mg·L^−1^) and RTE (50%) which results in their decolorization. This is in agreement with the previous reports, where induction and involvement of the enzymes laccase, tyrosinase, veratryl alcohol oxidase, azoreductase and DCIP reductase in the metabolism of azo dyes by bacteria has been shown [17,34,56,57,58].

**Table 3:** Enzyme activities in microbial consortium control at 0 h (before addition of dye or textile effluent) and after CR dye (100 mg·L^−1^) and RTE (50%) additions.

Enzymes	Control (0 h)	After Decolorization of CR Dye (12 h)	After Decolorization of RTE (20 h)
Laccase ^1^	0.4 ± 0.02	1.5 ± 0.02 ***	1.8 ± 0.02 ***
Tyrosinase ^2^	1.0 ± 0.01	2.1 ± 0.03 **	2.7 ± 0.03 **
Veratryl alcohol oxidase ^3^	1.3 ± 0.03	1.9 ± 0.04 **	2.3 ± 0.04 **
Azoreductase ^4^	0.4 ± 0.02	3.2 ± 0.03 ***	3.8 ± 0.03 ***
DCIP reductase ^5^	9.0 ± 1.6	38.6 ± 2.0 ***	42.8 ± 2.2 ***

Values are a mean of three experiments ±SE. Significantly different from control cells at ** *p* < 0.01 and *** *p* < 0.001 by one-way ANOVA with Tukey-Kramer comparison test. ^1^ µM of ABTS oxidized min^−1^·mL of enzyme^−1^·mg of protein^−1^; ^2^ µM of catechol oxidized min^−1^·mL of enzyme^−1^·mg of protein^−1^; ^3^ µM of veratryl alcohol oxidized min^−1^·mL of enzyme^−1^·mg of protein^−1^; ^4^ μM of Methyl red reduced min^−1^·mL of enzyme^−1^·mg of protein^−1^; ^5^ μM of DCIP reduced min^−1^·mL of enzyme^−1^·mg of protein^−^^1^.

### 3.6. Analysis of Dye CR Degradation

**Figure 6:**
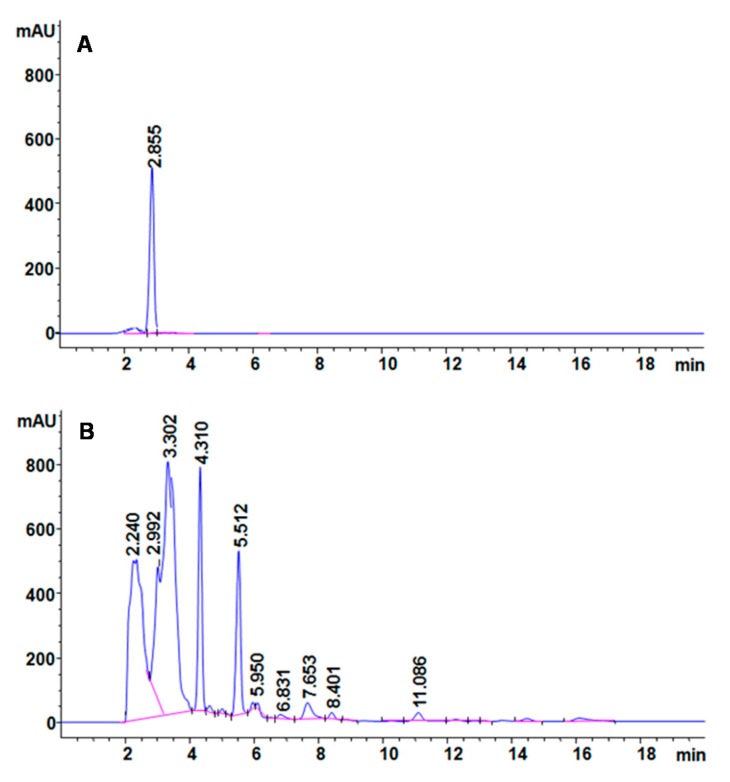
HPLC profile of (**A**) CR dye and (**B**) its decolorized products obtained after treatment with PUF-immobilized microbial consortium in an upflow column bioreactor.

The mechanism behind decolorization of CR azo dye by the PUF-immobilized microbial consortium in an upflow column bioreactor was analyzed with different analytical techniques. The HPLC spectra of CR dye control showed a single peak at a retention time of 2.855 min (Figure 6A). After treatment with PUF-immobilized microbial consortium in an upflow column bioreactor, the dye control peak disappeared and ten new peaks with different retention times of 2.240, 2.992, 3.302, 4.310, 5.512, 5.950, 6.831, 7.653, 8.401 and 11.086 min appeared (Figure 6B). The disappearance of the control dye peak and appearance of several new peaks in the treated samples suggests the biotransformation of dye CR into different metabolites.

**Figure 7:**
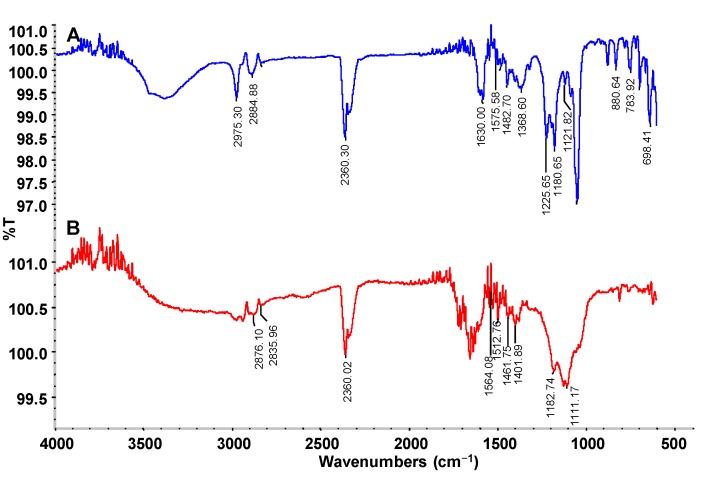
FTIR profile of (**A**) CR dye and (**B**) its decolorized products obtained after treatment with PUF-immobilized microbial consortium in an upflow column bioreactor.

FTIR analysis of the dye control and its products after decolorization by the PUF-immobilized microbial consortium in an upflow column bioreactor was carried out to check the changes in functional groups. The FTIR spectral peaks 2975.30 and 2884.88 cm^−1^ observed in the CR dye control represent C–H stretching as in alkanes (–CH_3_–) while the 2360.30 cm^−1^ band represents NH^+^ stretching as in charged amines (C=NH^+^) (Figure 7A). The specific peaks at 1630.00 and 1575.58 cm^−1^ indicate the presence of azo –N=N‒ double bond stretching as in azo compounds. In addition, the 1482.70, 1368.60, 1225.65, 1180.65 and 1121.82 peaks represent C–H deformation of alkanes, CH_3_ deformation of tertiary butyl symmetric CH_3_ bending, skeletal stretching of alkanes and C–N vibration of aliphatic amines, respectively. The 880.64 peak corresponds to the C–H deformation of a tetra or penta-substituted benzene containing one free hydrogen.

On the other hand, the FTIR spectra of the dye products decolorized by the microbial consortium showed a significantly different pattern of absorption peaks when compared to the parent CR dye. The specific azo bond peaks at1630.00 and 1575.58 cm^−1^ disappeared after microbial consortium treatment, indicating the reductive breakdown of the CR azo dye (Figure 7B). Additionally, the peaks from 880.64 to 698.41 cm^−1^ representing the benzene ring structures of the parent CR dye also disappeared suggesting the cleavage of the dye molecule. Overall, the FTIR spectra of CR dye control differed significantly from the products decolorized by the microbial consortium in the upflow column bioreactor, supporting the biotransformation of the dye into different metabolites.

GC–MS analysis data was used to determine the probable metabolites produced and also to propose a degradation pathway of CR dye by PUF-immobilized microbial consortium. The structures of detected metabolites were assigned from the fragmentation pattern and *m/z* values obtained (Table 4). Result of the mass spectrum analysis revealed the presence of three intermediate CR dye degradation metabolites, *viz*. biphenyl-1,4′-diamine, biphenyl and naphthalene.

**Table 4:** GC–MS analysis data of degraded products of dye CR by PUF immobilized microbial consortium under upflow column bioreactor.

Mass Spectrum	Name of the Product	Mol. Weight (*m/z*)
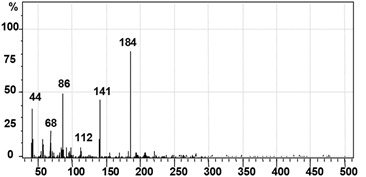	Biphenyl-1,4′-diamine [A]	184 (*m/z* = 186) (−2)
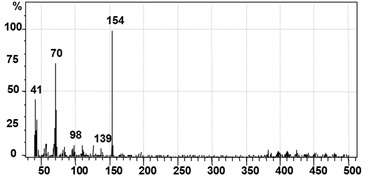	Biphenyl [B]	154 (*m/z* = 156) (−2)
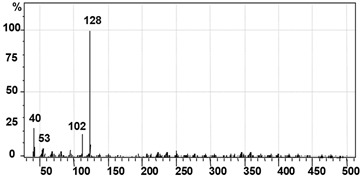	Naphthalene [C]	128 (*m/z* = 129) (−1)

**Figure 8:**
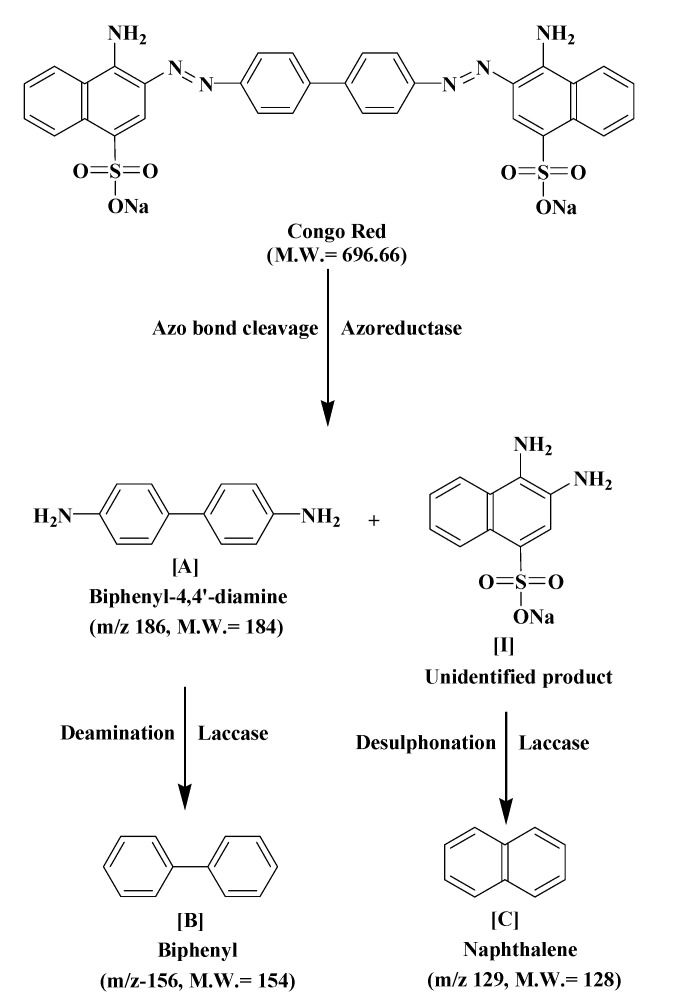
The proposed biodegradation pathway of CR dye by PUF-immobilized microbial consortium in an upflow column bioreactor.

Figure 8 depicts the proposed biodegradation pathway of CR dye by PUF-immobilized microbial consortium in an upflow column bioreactor. The possible mineralization of CR dye starts with the initial conversion of the dye to form biphenyl-1,4′-diamine [A] and unidentified intermediate [I] catalyzed by azoreductase that cleaves the azo bond. The initial step in the microbial degradation of azo dyes is cleavage of electrophilic azo bonds which leads to their decolorization [59]. This cleavage is known to form aromatic amines as end products; however most such amines further get degraded by the actions of oxidative enzymes [60]. The disappearance of the FTIR spectral peaks at 1630.00 and 1575.58 cm^−1^ in the treated dye metabolites support the azo bond cleavage of CR dye as shown in the proposed pathway.

It has been shown that a bacterial consortium consisting of *Providencia rettgeri* strain HSL1 and *Pseudomonas* sp. SUK1 performed the reduction of azo dyes under microaerophilic incubation conditions to produce toxic aromatic amines, but these disappeared in a subsequent aerobic process due to the activities of oxidative enzymes [61]. Similarly, in the present study the metabolite [A] undergoes deamination by the oxidative enzyme laccase to form biphenyl [B], while intermediate [I] udergoes desulphonation, also by the laccase, to form the low molecular weight compound naphthalene [C] as final dye degradation products. The FTIR spectral peaks from 1564.08–1390 cm^−1^ in the decolorized dye samples support the presence of naphthalene as shown in the proposed pathway. The naphthalene ring stretching vibrations are expected in the 1620–1390 cm^−1^ infrared region [62]. The oxidative cleavage of metabolite [B] and intermediate [I] are supposed to be caused by laccase activity, which is known to cleave the dye molecules asymmetrically [63]. Additionally, considerable induction in the activities of tyrosinase and veratryl alcohol oxidase suggested their involvement in the oxidation of metabolite [A] and [I]. Lade *et al.* during their study on biodegradation of the azo dye Trypan blue by an nriched microbial consortium reported that the dye was enzymatically degraded to form the low molecular weight compound naphthalene-1-ol as final intermediate metabolite [22].

### 3.7. Toxicity Analysis

Although the present PUF-immobilized microbial consortium treatment in an upflow column reactor was efficient in the complete decolorization and degradation of the azo dye CR and RTE, it is essential to evaluate the toxicity of the treated samples. Treated dye wastewaters are mostly released to nearby farms for irrigation purposes and thus have a direct impact on the growth of crop plants as plant survival under dye stress is stunted. The evaluation of toxicity in terms of seed germination and plant survival is therefore extremely important for determining the suitability of treated wastewaters for agricultural applications. Phytotoxicity analysis of dye and textile effluent using seedlings of common agricultural crops has been suggested as the primary toxicity test to assess the toxic nature of dyes and their degradation metabolites [64]. Results of the phytotoxicity test showed that the germination rate of seeds exposed to both untreated CR dye (100 mg·L^−1^) and RTE (50%) was inhibited by 80% and 90% in *S. vulgare* and 80% and 90% in *P. mungo*, respectively (Table 5). At the same time, less elongation in length of shoots *i.e.*, 4.1 and 4.2 cm for dye and 3.8 and 4.1 cm for textile effluent, as well as roots *i.e.*, 2.2 and 1.2 cm for dye and 2.0 and 0.9 cm for textile effluent, were also observed among both plants *S. vulgare* and *P. mungo*, respectively. The lower elongation in roots and shoots of both the plants exposed to untreated dye and textile effluent indicate a toxic effect on plant growth. In contrast, 100% germination of both the seeds with 11.2 and 6.7 cm of shoot and root length for *S. vulgare* and 14.3 and 6.2 cm of shoot and root length for *P. mungo* were found. It was also observed that seeds exposed to treated dye wastewaters showed similar germination as well as shoot and root lengths as like that of distilled water. These results clearly indicate that the treated dye and textile effluent samples were almost as non-toxic as distilled water. Moreover, the plants grown with treated samples were healthy in terms of shoot and root lengths, suggesting the conversion of complex dyes into simple oxidizable forms of a non-toxic nature.

**Table 5:** Phytotoxicity analysis of control CR dye (100 mg·L^−1^), RTE (50%) and their treated samples using PUF-immobilized microbial consortium in an upflow column bioreactor.

Samples	*S. vulgare*			*P. mungo*		
	Germination (%)	Shoot Length (cm)	Root Length (cm)	Germination (%)	Shoot Length (cm)	Root Length (cm)
Distilled water	100	11.2 ± 0.3	6.7 ± 0.2	100	14.3 ± 0.3	6.2 ± 0.3
CR (100 mg·L^−1^)	20	4.1 ± 0.1 *	2.2 ± 0.2 *	20	4.2 ± 0.3 *	1.2 ± 0.2 *
CR metabolites	90	10.1 ± 0.2 ^$^	5.6 ± 0.3 ^$^	80	14.1 ± 0.2	5.9 ± 0.2 ^$^
RTE (50%)	10	3.8 ± 0.2 *	2.0 ± 0.2 *	10	4.1 ± 0.2 *	0.9 ± 0.1 *
RTE metabolites	80	9.9 ± 0.2 ^$^	5.4 ± 0.2 ^$^	70	13.9 ± 0.2	5.5 ± 0.2 ^$^

Values are a mean of three experiments ±SE. Root and shoot lengths of plants grown with CR dye solution (100 mg·L^−1^) and RTE (50%), respectively, were significantly different from that of plants grown in distilled water (* *p* < 0.1). Root and shoot lengths of plants grown with the treated CR dye and RTE, respectively, are also significantly different from that of plants grown in dye CR solution and RTE (^$^ *p* <0.1). The significance was analyzed by one-way ANOVA with Tukey-Kramer multiple comparison test.

Acute toxicity testing has been described for the intoxication of mammals by chemical consumption. The mortality test with aquatic crustacean *D. magna* is the most common and rapid acute toxicity assay used to assess the toxic effect of chemicals on mammals, including human beings [65]. Results of the acute toxicity assay showed complete mortality of *D. magna* exposed to CR dye (100 mg·L^−1^) solution, as well as RTE (50%), whereas only 10% and 20% mortality was observed in treated dye and textile effluent samples, respectively (Table 6). Similar results were reported by Lade *et al.*, where complete mortality of *D. magna* was observed in the presence of untreated azo dyes [61]. The complete mortality of *D. magna* in untreated dye and textile effluent indicates their toxic nature, while treated samples showed greater crustacean survival. The results of both the phytotoxicity and acute toxicity assays with dye and textile effluent thus confirm the less toxic nature of the treated samples. The detoxification of dye and textile effluent might be due to removal of color and reduced COD levels. This clearly suggests the possible use of PUF-immobilized microbial consortium upflow column bioreactors for the mineralization and detoxification of azo dyes.

**Table 6:** Mortality of *D. magna* exposed to CR dye (100 mg·L^−1^), RTE (50%) and their culture supernatants obtained after treatment with PUF immobilized microbial consortium in an upflow column bioreactor.

Samples	Mortality (%)
Distilled water	0 ± 0.0
CR (100 mg·L^−1^)	100 ± 0.0
CR treated broth supernatant	10 ± 0.0
RTE (50%)	100 ± 0.0
RTE treated broth supernatant	20 ± 0.0

Values are a mean of three experiments ±SE.

## 4. Conclusions

This study explores an upflow column bioreactor using WB as microbial growth medium for low-cost mineralization of azo dyes and textile effluents. The result of the batch decolorization study together with demonstration of an upflow column bioreactor configuration established the potential of PUF-immobilized microbial consortium in dye removal. The upflow column bioreactor showed efficient decolorization of CR dye and RTE with a significant reduction in TOC, COD and BOD contents. Induction of the activities of oxido-reductive enzymes suggested their role in the decolorization process. Besides mineralization, the toxicity of both dye and textile effluent was also decreased after treatment. Thus, the overall dye removal performance by PUF-immobilized microbial consortium in an upflow column bioreactor offers new insight into the use of WB as growth medium for low-cost bioremediation of azo dyes at a large scale.
